# Nanobodies As Tools to Understand, Diagnose, and Treat African Trypanosomiasis

**DOI:** 10.3389/fimmu.2017.00724

**Published:** 2017-06-30

**Authors:** Benoit Stijlemans, Patrick De Baetselier, Guy Caljon, Jan Van Den Abbeele, Jo A. Van Ginderachter, Stefan Magez

**Affiliations:** ^1^Laboratory of Cellular and Molecular Immunology, Vrije Universiteit Brussel (VUB), Brussels, Belgium; ^2^Myeloid Cell Immunology Lab, VIB-UGent Center for Inflammation Research, Ghent, Belgium; ^3^Laboratory of Microbiology, Parasitology and Hygiene (LMPH), University of Antwerp (UA), Antwerp, Belgium; ^4^Unit of Veterinary Protozoology, Department of Biomedical Sciences, Institute of Tropical Medicine Antwerp (ITM), Antwerp, Belgium; ^5^Laboratory for Biomedical Research, Ghent University Global Campus, Incheon, South Korea

**Keywords:** nanobody, diagnosis, treatment, African trypanosomes, paratransgenesis

## Abstract

African trypanosomes are strictly extracellular protozoan parasites that cause diseases in humans and livestock and significantly affect the economic development of sub-Saharan Africa. Due to an elaborate and efficient (vector)–parasite–host interplay, required to complete their life cycle/transmission, trypanosomes have evolved efficient immune escape mechanisms that manipulate the entire host immune response. So far, not a single field applicable vaccine exists, and chemotherapy is the only strategy available to treat the disease. Current therapies, however, exhibit high drug toxicity and an increased drug resistance is being reported. In addition, diagnosis is often hampered due to the inadequacy of current diagnostic procedures. In the context of tackling the shortcomings of current treatment and diagnostic approaches, nanobodies (Nbs, derived from the heavy chain-only antibodies of camels and llamas) might represent unmet advantages compared to conventional tools. Indeed, the combination of their small size, high stability, high affinity, and specificity for their target and tailorability represents a unique advantage, which is reflected by their broad use in basic and clinical research to date. In this article, we will review and discuss (i) diagnostic and therapeutic applications of Nbs that are being evaluated in the context of African trypanosomiasis, (ii) summarize new strategies that are being developed to optimize their potency for advancing their use, and (iii) document on unexpected properties of Nbs, such as inherent trypanolytic activities, that besides opening new therapeutic avenues, might offer new insight in hidden biological activities of conventional antibodies.

## Introduction

African trypanosomiasis (AT), caused by strictly extracellular unicellular flagellated protozoan parasites belonging to the genus *Trypanosoma*, is a “neglected” disease of medical and veterinary importance that significantly affects the socioeconomic development of sub-Saharan Africa (1–5). Hereby, AT affects mainly remote rural areas with minimal health infrastructure and its distribution coincides mostly with the habitat of the hematophagous insect vector, i.e., the tsetse fly (*Glossina* sp.) (6). In humans, the disease is known as human African trypanosomiasis (HAT) or sleeping sickness, and is caused by (i) *Trypanosoma brucei gambiense* (Western and central Africa) which is an anthroponotic disease with a minor role for animal reservoirs accounting for 98% of the reported HAT cases, and causing a chronic, gradually progressing disease with limited symptoms, whereby the late meningoencephalitic stage is reached after months/years of infection (7–9), and (ii) *Trypanosoma brucei rhodesiense* (Eastern/southern Africa) which is a zoonotic disease affecting mainly animals (livestock and wildlife), with humans being only occasionally infected, and representing 2% of the reported HAT cases, whereby the infections are more acute and virulent/lethal with a rapid progression (within weeks) to the late meningoencephalitic stage (3, 9–12). Hence, the zoonotic nature of *T. b. rhodesiense* infections makes them more difficult to control compared to *T. b. gambiense* infections (8, 13, 14). Animal African trypanosomiasis (AAT) or Nagana is the second form of AT affecting sub-Saharan Africa, which is mainly caused by *Trypanosoma congolense, Trypanosoma vivax*, and to a lesser extent *Trypanosoma brucei brucei*, whereas Surra and Dourine are also forms of AAT caused by *Trypanosoma evansi* and *Trypanosoma equiperdum*, respectively (15–17). Overall, *T. congolense* is a major constraint for livestock production in sub-Saharan Africa, whereby cattle succumb to infection primarily due to parasite-induced anemia or complications resulting from secondary, opportunistic infections (18). In addition, the estimated annual losses associated with AAT are about US$5 billion (1, 19–21), which is mainly due to a combined result of political, sociocultural, environmental, entomological, and livestock management factors (22–24). So far, chemotherapy is the only strategy available to treat the disease, whereby unique organelles of trypanosomes (glycosomes or kinetoplast) that are absent in the mammalian host or trypanosome metabolic pathways that differ from their host counterparts (carbohydrate metabolism, protein and lipid modifications, and programmed cell death) are targeted (25–27). Given that chemotherapy is associated with high drug toxicity, there is an urgent need to optimize trypanocide usage and delivery in order to decrease the risk of toxicity and/or resistance development (28–30). Control of AT is also hampered due to inefficient diagnosis of the infection especially for AAT and *T. b. rhodesiense* HAT where microscopical parasite detection (cheap but with low sensitivity), detection of the parasite’s DNA (expensive but with high sensitivity), or anti-parasite antibodies remain the only available tools for diagnosis. Yet, these techniques require specialized equipment and personnel and hence are not suitable for direct use in the field. Only for *T. b. gambiense*, monitoring tools are available for both detection and staging of the disease (4, 31–33). Existing field applicable antibody-based diagnostic tests still suffer from a lack of positive predictive value and cannot differentiate between active or cured infections (32, 34, 35). Direct diagnosis aimed at parasite antigen detection is often hampered by sequestration of parasite antigens by the host’s antibodies or by concealing of epitopes from the diagnostic monoclonal antibodies (mAbs) (36). Although immunodiagnostics based on antigen detection would be preferable, they are currently not available for trypanosomiasis in the field (32).

In contrast to conventional antibodies, nanobodies [Nbs or VHHs, i.e., camelid-derived single-domain antibody fragments (~15 kDa) that are selected through phage display technology and panning methodologies] (37, 38) could be used to overcome certain challenges faced by mAb-based tests (see above). Hereby, Nbs exhibit characteristic features such as (i) a nanomolar affinity for their target (39), (ii) a unique epitope recognition spectrum different from conventional antibodies, thereby allowing detection of both free antigens and those bound by host antibodies (40), (iii) high solubility (40), (iv) easy tailorability (multimerization or tagging) for molecular imaging and drug-delivery applications (41–44), and (v) small size that circumvents problems of tissue or blood–brain barrier (BBB) penetrability (44, 45). Due to these unique biochemical and biophysical properties, they are considered as promising next-generation therapeutics with great potential in pharmaceutical and industrial applications (46, 47). Indeed, Nbs are increasingly exploited in protein structure/function studies and in the development of alternative or new medical diagnostic and therapeutic applications (48, 49). Nbs also possess a relatively high thermostability (50, 51) and are consequently attractive for the development of immunodiagnostic tests that could be applicable in hot climatic conditions (i.e., sub-Saharan Africa).

In the next sections, we will give an overview of how the Nb technology can be implemented in the fight against AT both at the level of diagnosis and treatment and finally how acquired knowledge on Nbs in AT might lead to new insights in the function of conventional antibodies in the immune system.

## Life Cycle of African Trypanosomes

**Figure 1:**
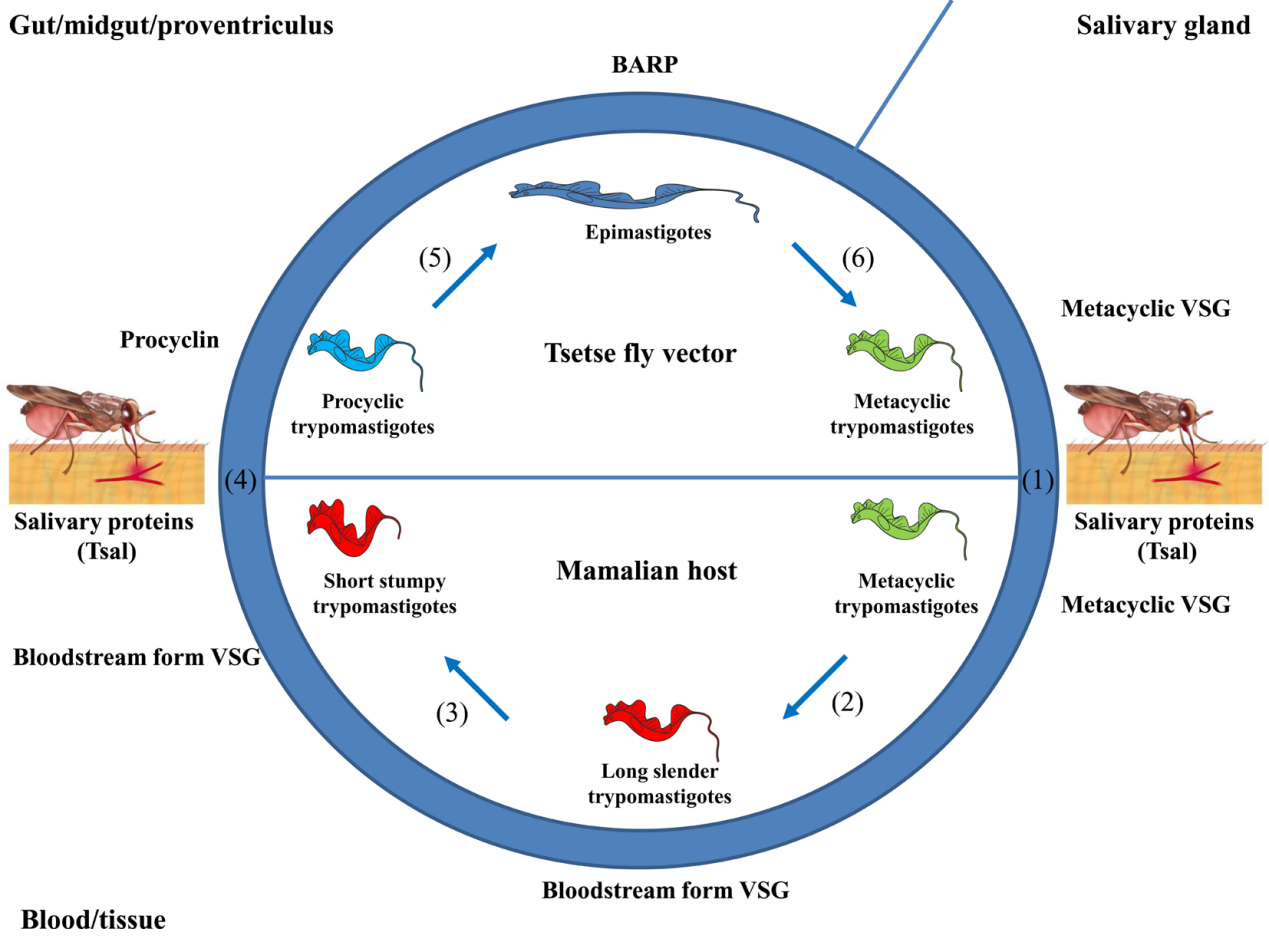
Life cycle of African trypanosomes. (1) Upon the bite of a trypanosome-infected tsetse fly, metacyclic parasites (trypomastigotes) and saliva components (such as Tsal) are inoculated into the mammalian host. (2) The metacyclic parasites [expressing a heterogeneous metacyclic variable surface glycoprotein (VSG)] differentiate into long slender (LS) trypomastigotes (i.e., LS, dividing/proliferating forms, expressing a unique bloodstream form VSG) giving rise to a first peak of parasitemia. (3) At the peak of parasitemia, these LS forms differentiate into non-dividing short stumpy (SS) forms that are pre-adapted to be taken up by the vector. (4) Upon taking a blood meal, these SS forms are ingested and in the midgut these parasites differentiate into procyclic forms (PF), whereby the coat is switched toward procyclin. (5) The PF differentiate into epimastigote forms when migrating to the proventriculus (expressing a bloodstream alanine rich protein coat). (6) Upon migration to the salivary glands, the parasites differentiate into metacyclic forms that are ready to complete their life cycle.

In order to point out at which stages Nbs might be applicable to fight AT, it is appropriate to overview briefly their life cycle. African trypanosomes exhibit a digenetic life cycle, alternating between the blood/tissues of the mammalian host and alimentary tract of the tsetse fly vector, whereby they exist as procyclic or trypomastigote forms (52), respectively (see Figure 1). The lifecycle within the mammalian host is initiated upon the bite of a trypanosome-infected tsetse fly when taking a blood meal (see Figure 1). Hereby, metacyclic parasites are inoculated in the host dermis in concert with tsetse saliva components that play a key role in the modulation of the host early immune response and in sculpturing an immune privileged microenvironment for infection initiation (53–55). From the dermal infection site, parasites reach the blood circulation through the lymphatics (55). Subsequently, these metacyclic parasites expressing a heterogeneous metacyclic variable surface glycoprotein (VSG) coat will differentiate into dividing long slender (LS) bloodstream dividing forms (BF), which express a unique VSG coat and are adapted to survive in the glucose-rich and highly oxygenated blood of the mammalian host. Next, these BFs rapidly multiply, giving rise to a first parasitemia peak. At the peak of parasitemia, most likely *via* a quorum sensing mechanism (56, 57), the LS parasites differentiate into non-dividing short stumpy (SS) forms pre-adapted for survival in the tsetse fly vector. Within the tsetse fly vector, these SS forms differentiate within the midgut into procyclic forms (PF) that express a procyclin coat, which are adapted to survive in the proline-rich (carbon source) and low-oxygenated environment. Within the tsetse fly, these parasites undergo several differentiation stages in the different parts of the alimentary tract, mouthparts, and salivary glands (58, 59). In order to adapt to the growth conditions imposed by the different environments of their hosts, trypanosomes undergo essential morphological and metabolic changes (52), consisting of fine-tuning their energy metabolism, a dedicated iron and nutrient uptake, organelle reorganization, and biochemical and ultrastructural remodeling (60–65). Hence, tools to interfere with the various stages in the parasite life cycle might be an attractive strategy to combat AT.

## Nbs as Versatile Tools for AT

The control over AT would benefit from more efficient diagnosis and treatment intervention strategies. Hereby, since their serendipitous discovery 30 years ago, Nbs attracted a progressively growing interest from fundamental research on antibody structure and ontogeny to diagnostical and therapeutical applications (66–68). With respect to fundamental research, the Nb technology was found to provide a novel tool in structural biology, whereby they can be used as crystallization-aid, or as a tool to design novel drugs based on co-crystallization of Nbs with their cognate antigen (69, 70). Although such applications in the field of AT are not yet documented, this will most likely become an emerging field of study in the near future. Moreover, given the tremendous efforts to identify novel targets for AT through proteomic approaches (71), the merging of the Nb technology with the current technologies might pave the way to develop additional tools/targets to fight AT. In this section, we will give an overview of the applications of the Nb technology in the field of AT.

### Nbs As Tools for Diagnosis

**Figure 2:**
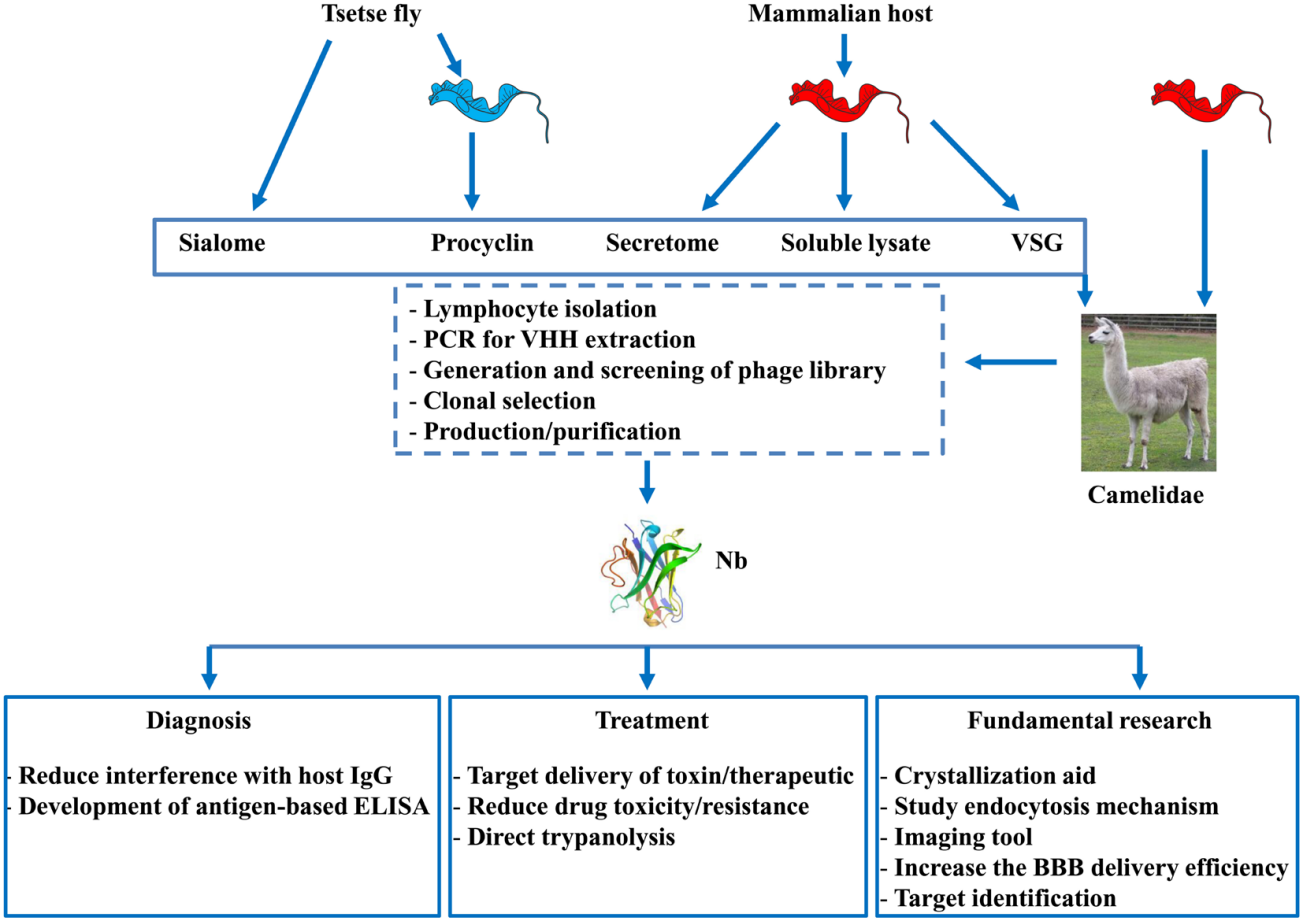
Overview of strategies used to generate nanobodies (Nbs) and their applications. Nbs can be obtained from llama’s that are either immunized with factors derived from (i) tsetse flies (i.e., sialome or procyclin coat isolated form procyclic forms) or (ii) purified blood stream parasites originally grown in the mammalian host [i.e., secretome, soluble lysate, or variable surface glycoprotein (VSG)] or (iii) from infected (naturally or experimentally) with trypanosomes. After immunization, lymphocytes are isolated from the blood and *via* the PCR, phage display, and different selection procedures, individual Nbs can be obtained and purified. These Nbs can find application in diagnosis, therapy, or be used for fundamental research aiming at developing novel strategies to fight African trypanosomiasis.

To date, several obstacles hamper an efficient and reliable diagnosis of AT, whereby (i) inefficient antibody-based detection due to interferences caused by the host’s antibody response (i.e., antibodies remaining in circulation for long periods of time) impairs discrimination between active and cured infections and (ii) antigen-based trypanosome detection methods that might allow circumventing the problems encountered in the antibody-based detection system are not yet available. In this context, Nbs are emerging as promising tools to overcome the limitations of antibody-based diagnosis and to improve antigen-based detection of African trypanosomes (see Figure 2 and Table 1). For instance, though the binding interference caused by the host’s antibody response upon infection might hamper sensitive detection, this response might also be exploited using the Nb technology. Indeed, Caljon et al. (72) showed that following immunization of an alpaca with the sialome of the savannah tsetse fly vector (*Glossina morsitans morsitans*), Nbs could be generated against an abundant highly immunogenic tsetse salivary gland (Tsal) endonuclease protein and could subsequently be used to monitor tsetse fly bite exposure (73). Monitoring this bite exposure level in a target host population is important as the probability of a trypanosome transmission event to a new host is directly linked to the exposure level of the host to tsetse bites in that area (=risk factor). Indeed, only a very limited number of flies in a natural tsetse population carries the final infective parasite stage, so the more frequent a host is bitten by tsetse the higher the risk is that it will trap a trypanosome infection through the bite of a rarely occurring infected fly in that area. The assay principle relies on the detection of specific anti-Tsal1 antibodies in an assay that measures the competitive binding onto the immunogenic Tsal1protein of diagnostic Nbs (Tsal1Nb-5 and -11) and host antibodies that are typically induced by bite exposure. This Nb-based competition assay allows specific detection of exposure to a range of important tsetse fly species in the context of sero-epidemiological surveys based on salivary proteins. This could not only allow monitoring/estimating the intensity of the host exposure to tsetse fly bites but also reveal the efficacy of applied and/or ongoing tsetse fly control activities. This approach might be further extended by generating anti-proteome and/or anti-infectome Nb libraries in order to identify diagnostic Nbs (74–76). Indeed, the work by Obishakin et al. (77) showed that upon immunization of a llama with *T. evansi* lysate, Nbs against the paraflagellar rod (PFR) protein of trypanosomes could be generated and used in a solid phase antigen-ELISA to detect this protein in different *T. evansi* strains. Although the assay was not sensitive enough to detect *T. congolense* and *T. vivax* lysates in ELISA, one of the anti-PFR Nbs (Nb392) was found to cross-react with multiple parasite species such as *T. brucei, T. congolense*, and *T. vivax* as well as PF. Detection was achieved using fixed and permeabilized parasites and *via* flow cytometry and immunofluorescence microscopy, inferring that this Nb could be used to develop a broad spectrum diagnostic reagent. Moreover, this Nb could also be exploited as a PFR marker and/or as a useful research tool to isolate PFR proteins. More recently, Odongo et al. (78) identified following immunization of a llama with the soluble proteome of bloodstream form (BF) *T. congolense* a Nb (Nb474) recognizing glycosomal aldolase (*Tco*ALD) that could be used in a Nb-based sandwich ELISA to specifically detect active *T. congolense* infections in experimentally and naturally infected cattle. In experimental *T. congolense* infection models, parasitemia and detected antigenemia followed the same trend and the assay was suggested to be suitable as a test of cure. Although no formal detection limit was determined, the Nb474-based test was able to detect *T. congolense* infections in two field collected cattle blood samples that underwent the traditional parasitological diagnosis using the buffy coat technique followed by 18S-PCR-RFLP-based parasite species identification. Furthermore, it was suggested that the robustness of this Nb474-ELISA to specifically monitor *T. congolense* infections in the field might be improved once the structural and biophysical determinants of the specific Nb474–*Tco*ALD interaction can be determined. Of note, also in other parasitic diseases such as malaria, aldolase was reported as a proficient biomarker for the detection of *Plasmodium vivax* (79). In addition, in regions of sub-Saharan Africa where animals are infected with human and animal infective trypanosomes, this selective test for *T. congolense* using aldolase as a biomarker would allow discriminating between the two parasite groups, hence enabling assessment of the potential risk for human infection (80).

**Table 1:** Overview of the different targets against which nanobodies (Nbs) have been generated.

Target	Vector	PF	BF	Specificity	Diagnosis	Treatment	Reference
Tsal	+	−	−	Saliva	+	−	(72)
Procyclin	−	+	−	**Tb**	?	?	**–**
Aldolase	−	−	+	**Tc**	+	?	(78)
Paraflagellar rod protein	−	+	+	**Te**, Tb, Tc, Tv	+	?	(77)
Conserved variable surface glycoprotein (VSG) epitope	−	−	+	**Tb**, (Tc?)	+	+	(81)
Variable VSG epitope	−	−	+	**Tb**, (Tc?)	+	+	(98)
Transferrin receptor	−	?	+	**Tb**, Tc, Te, Tv	?	?	–

*In addition, the Nb specificity and applicability (diagnosis/treatment) is mentioned*.

*PF, procyclic forms; BP, bloodstream form; Tb, Trypanosoma brucei; Tc, Trypanosoma congolense; Te, Trypanosoma evansi; Tv, Trypanosoma vivax*.

*Trypanosome species in bold refers to the species against which the target was originally generated*.

*“?” stands for not tested or unknown*.

Besides recognizing low abundant proteins, Nbs have also been generated against the highly abundant VSG coat of the *T. congolense* parasite, yet due to the system of antigenic variation their diagnostic value is rather limited (unpublished data). However, the work by Stijlemans et al. (81), using the *T. brucei* model parasites, showed that the reduced size of a Nb allows to target conserved, less-immunogenic, cryptic VSG epitopes, that are inaccessible to conventional antibodies. Hereby, the fluorescently labeled anti-VSG Nb-33 was found to be very proficient to detect different isoforms of the *T. brucei* family and to specifically stain trypanosomes in infected blood. This suggests that even Nbs directed against specific conserved regions of the VSG molecule can be used as diagnostic tools.

Collectively, these data suggest that a Nb-based strategy could be a unique approach for diagnosis development and might bring us a step closer toward obtaining an antigen-detection test that can be used for rapid and reliable detection of vector exposure as well as the presence of pathogen infections in reservoir hosts (see Figure 2 and Table 1). In addition, Nbs could also be used for target discovery given that following immunizations with complex protein mixtures and *via* different purification techniques, their cognate antigens with diagnostic potential can be identified.

### Nbs As Therapeutic Devices

#### Nbs As Tools for Drug/Toxin Delivery

Although the currently used chemotherapeutics are proficient in killing trypanosomes, their *in vivo* application suffers from systemic drug toxicity and occurrence of drug resistance (82). Therefore, delivering the chemotherapeutic (or toxin) directly to the parasite could be a more efficient way for treating AT. The possibility of using Nbs as targeting entity was investigated using the Nb-33 as model Nb (Figure 2; Table 1). With respect to using Nb-33 as a *toxin-delivery system* to African trypanosomes, apolipoprotein L-1 (ApoL-1) was selected as a trypanolytic component. ApoL-1 is a component of normal human serum (NHS) that exerts a direct trypanolytic effect on all AAT species, except resistant forms such as *T. brucei rhodesiense* (83). Indeed, *T. b. rhodesiense* expresses the apoL-I-neutralizing serum resistance-associated (SRA) protein, endowing this parasite with the ability to infect humans and cause HAT (83, 84). Hence, Nb-33, recognizing a conserved/cryptic region within the VSG coat, was coupled to a truncated form of Apo-L1 (i.e., Tr-apoL-1), which is engineered by deleting the SRA-interacting domain. This engineered immunotoxin was shown to function curatively and to alleviate effects on acute and chronic infections in mice infected with both NHS-resistant and sensitive parasites (85, 86). The Nb-33 was also used as a *delivery system for therapeutics* using *T. b. gambiense* parasites as a model. Hereby, nanoparticles loaded with pentamidine [i.e., drug used for treating the early disease stage, before central nervous system involvement (87), as the second-line option to suramin] were coupled to Nb-33. This targeted drug-bullet allowed decreasing the half-inhibitory concentration (IC50) 7-fold compared to free drug *in vitro* and cured all mice at a 10-fold lower dose than the minimal full curative dose of free pentamidine (88–90). Moreover, recently an improved version of this nanocarrier allowed reducing the curative dose 100-fold and circumvented drug resistance that is due to mutations in aquaglyceroporin 2 (i.e., the surface channel protein that mediates pentamidine uptake in *T. brucei*) (29, 91, 92). Overall, targeting the trypanosome surface using Nb-coated drug-loaded nanoparticles can be an elegant way to deliver drugs *via* endocytosis and bypass the usual drug delivery route altogether. Recently, using the Nb-33, it was shown that Nbs are able to pass the BBB during the late stage of *T. brucei* infection using murine and rat models, which suggests they might be valuable tools to target toxins even at the levels of the BBB. Yet, they were incapable of accumulating in the brain at therapeutically relevant concentrations (93), which is most likely due to the systemic pharmacokinetics of monovalent Nbs (42, 94). Hence, efforts should be undertaken to tailor these Nbs *via* for instance CDR grafting to yield improved brain penetrating properties without losing their beneficial size (95, 96). In addition, to quantitatively study the penetration of Nbs into the CNS *in vivo*, intracerebral microdialysis represents a powerful and sensitive technique. Given that much effort is put into developing new drugs against African trypanosomes, the Nb-based drug/toxin-delivery approach might allow increasing their efficacy, even against drug resistant parasites, and be applicable for many other diseases (97).

#### Nbs As Direct Trypanolytic Entities by Blocking Endocytosis

By serendipity, besides a drug-targeting potential, Nbs were found to exert direct trypanolytic activities. Indeed, it was shown that some Nbs specific for the variable part of VSG of *T. b. brucei* parasites were able to induce parasite lysis *in vitro*. The lytic process consists of a rapid immobilization of the parasites, followed by massive enlargement of the flagellar pocket and a major blockage of endocytosis (98). Given that endocytosis is essential for trypanosome survival, playing a key role in nutrient uptake and in regulating intracellular ATP-levels as well as in maintaining the mitochondrial membrane potential, it is a prime candidate target for therapeutic interventions (99). Moreover, the endocytosis is confined to the flagellar pocket, which can be considered as the gateway to and from the cell surface that regulates the host–parasite interface as well as significantly contributes toward interactions with therapeutics. Hence, this might be the trypanosomal Achilles’ heel and offer perspectives for directed drug delivery focusing on proteins essential in endocytosis (100). Also, targeting specific receptors essential for nutrient uptake could be considered as therapeutic targets. For instance, given that iron is essential for the survival of both the trypanosome and the mammalian host and that their receptor for iron/transferrin uptake differs (i.e., a heterodimer and homodimer, respectively) (101), specific targeting of the parasite transferrin receptor using Nbs might be an opportunity to selectively deprive trypanosomes from this essential nutrient or alternatively be used as drug delivery or diagnostic tool. Also, the fact that low-molecular weight VSG-specific trypanolytic Nbs can impede endocytosis suggests that Nbs can be used as tools to further unravel the fascinating endocytosis mechanism used by trypanosomes. This in turn may offer new opportunities for developing novel trypanosomiasis therapeutics aimed at affecting endocytosis.

One aspect that could compromise the therapeutic applications of Nbs, in for instance HAT patients, is the potential immunogenicity of Nbs, especially in treatments that require repeated injections. Currently, the immunogenicity of Nb-based therapeutic applications is controversial. For instance, a clinical trial study conducted by GSK revealed the occurrence of anti-TNFR1 Nb autoantibodies and in another study with a tetravalent anti-DR5 receptor Nb hepatotoxicity in patients with such pre-existing antibodies has been described (102, 103). By contrast, no anti-HER2 Nb autoantibodies could not be detected in patients who received a non-humanized Nb (104), nor in patients receiving a Nb against von Willebrand factor (105). Also in the murine model, so far, multiple injections of Nbs in different disease settings did not result in immunogenicity, neither at the level of specific antibodies against Nbs nor at the level of cell proliferation and cytokine levels (68, 106–108). Hence, it seems that the occurrence of immunogenicity might depend on the target and disease situation (46). Yet, to reduce/minimize the risk of an immune response within the mammalian host, there are strategies currently implemented, such as humanization of the Nbs, whereby the camelid-specific amino acid sequences are mutated to their human heavy chain variable domain equivalent. In this context, a universal humanized Nb scaffold has been generated that allows grafting the antigen-binding loops from other Nbs, thereby transferring the antigen specificity and affinity (109).

#### Paratransgenesis As Tool to Deliver Nbs within the Tsetse Fly Vector

The applications of Nbs against AT may be not restricted to the BF of the parasites within the mammalian host, but could be applied/extrapolated to the vector. In this context, the possibility to exploit the tsetse fly bacterial symbiont *Sodalis glossinidius* as a paratransgenic platform organism for the expression and delivery of trypanosome-interfering proteins (i.e., Nbs) within the tsetse fly vector was evaluated. To this end, both the non-lytic Nb-33 and the trypanolytic Nbs were shown to be successfully expressed without affecting *S. glossinidius* fitness/viability (110). Moreover, using the trypanolytic Nb as proof of concept, recombinant *S. glossinidius* could settle in different tsetse fly tissues at high densities. Furthermore, significant levels of functional anti-trypanosome Nbs were released in several tissues including the midgut where important developmental stages of the parasite reside (111). Here, the level of Nb expression was estimated to be in the low nanogram (<10 ng), which was calculated to be sufficient to lyse the expected low number of transforming blood stream trypanosomes (around 10^3^ parasites) in the tsetse midgut during the early developmental period after ingestion by the fly (111). Accordingly, this paratransgenic approach using *Sodalis* to deliver Nbs that target the parasite or the trypanosome–tsetse fly cross talk could open new avenues to unravel the molecular determinants of this specific parasite–vector interplay and to ultimately render tsetse flies trypanosome resistant [reviewed by Caljon et al. (112)]. Given that the trypanosome is not exposed to an adaptive immune system in the tsetse vector, this parasite stage is not undergoing antigenic variation with the major surface antigen being encoded by a limited set of procyclin genes. In this context, the potential of Nbs delivered using the *Sodalis* endosymbiont targeting the major developmental stages in the tsetse fly, such as the procyclic trypanosomes that need to overcome the midgut barrier in order to achieve colonization of the tsetse fly vector, is currently being investigated. Stable integration of Nb expression cassettes in the *Sodalis* genome (e.g., by using recently established procedures) and efficient vertical transfer of the transgenic *Sodalis* have been achieved (113). Important issues, such as the identification of highly potent infection-blocking Nbs and the increased proteolytic stability, are still to be addressed (114). The latter feature will be highly beneficial to maintain potent effector levels in the strong proteolytic digestive environment of the insect midgut. Yet, such strategies could potentially culminate in a drug-targeting strategy to eliminate trypanosomes within the tsetse fly vector. An important achievement in the context of the *Sodalis*-based paratransgenesis is the efficient transfer of genetically modified *Sodalis* from the mother tsetse fly to its offspring through intrauterine nourishment (113). This implies that a large-scale tsetse fly colony can be established of flies harboring a Nb-expressing *Sodalis*. The paratransgenic trypanosome-resistant male flies from these colonies can then be released (after sterilization through irradiation) at a massive scale in the context of the Sterile Insect Technique (SIT). SIT was already successfully used to eradicate the tsetse fly in Zanzibar and is currently an important pillar in the tsetse fly control campaigns in Ethiopia and Senegal (http://www.fao.org/in-action/senegal-celebrates-first-victory-against-tsetse-fly-eradication/en/).

## Lytic Nbs: Relevance *In Vivo*?

The observation that the antigen-binding domain (i.e., Nb) on itself, in the absence of the Fc part can exert a significant Fc-independent killing of African trypanosomes *in vitro* and *in vivo* is remarkable (98). Moreover, it was found that both the size and affinity were of crucial importance for this trypanolytic activity (98, 115). Indeed, whereas polyclonal antibodies (including heavy-chain antibodies) specific for the VSG of African trypanosomes are completely harmless to trypanosomes in the absence of complement or any other bystander effector (116, 117), polyclonal Fabs or Nbs derived from the serum antibody pools and monoclonal/polyclonal Fabs or Nbs that are deprived of all effector functions (i.e., Fc) could exhibit an intrinsic trypanolytic activity *in vitro* (98). It is surprising that removal of the Fc part from antibodies unveils a novel but deadly situation, because trypanosomes and other extracellular pathogens mostly coped during evolution with intact immunoglobulins and thus developed multiple ways to avoid the destructive action of such large molecules (118, 119). In case of African trypanosomes such escape mechanisms include (i) antigenic variation of the VSGs, (ii) dense packing of VSG molecules on the parasite’s coat prohibiting the recognition of conserved and/or physiologically important epitopes by intact antibodies, (iii) clearing of VSG-bound antibodies by endocytosis of the VSG–antibody complex [reviewed in Ref. (120–123)]. In contrast to intact Abs, the small-sized Fabs or Nbs may penetrate the dense VSG coat and trigger new processes or avoid removal of VSG–antibody complexes, which is dictated by the bivalency of Abs (possibly due to cross-linking of the VSGs) and/or antibody size, e.g., whereby the presence of the Fc part leads to steric occlusion (123, 124).

**Figure 3:**
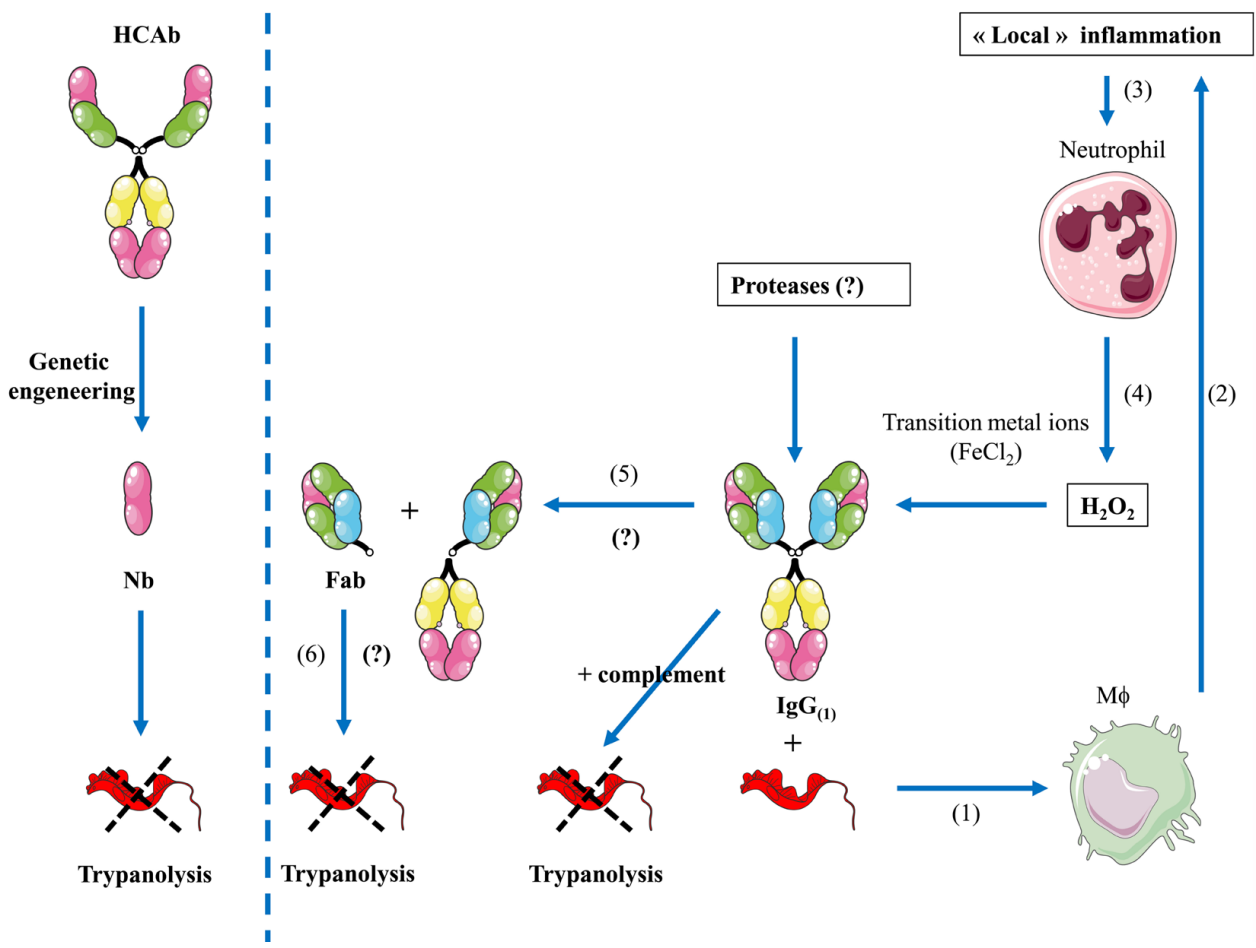
Proposed model of “hidden” functions of antibodies during inflammation. (Left panel) Through genetic engineering, camelid-derived heavy-chain antibodies (HCAbs) can be fragmented to the size of a nanobody that might exert a direct effect on pathogens (i.e., trypanosomes). (Right panel) (1) During infection (i.e., trypanosome infection), parasite-derived factors in concert with host-derived factors trigger macrophage (Mϕ) activation. (2) Hereby, elicited antibodies opsonize parasites and in concert with complement trigger parasite destruction, further leading to release of macrophage activating components and inflammatory responses. (3) The local inflammation may lead to recruitment, activation, and apoptosis of neutrophils. (4) Release of H_2_O_2_ as well as transitional metal ions and proteases by neutrophils/activated macrophages can fragment intact IgG (1). (5) Next, this will induce hinge fragmentation of IgG1 giving rise to a fragmented antibody into moieties including the Fab domain. (6) This Fab domain might exert a direct lytic effect on trypanosomes.

The concept that the Fc part within an antibody is masking the intrinsic destructive capacity of the antigen-binding fragment is intriguing. Consequently, other polyclonal or mAbs may share similar features and harbor hidden activities that remain occluded within intact Abs and might manifest themselves upon generation of monovalent Fabs or Nbs (see Figure 3). Evidence for a possible intrinsic anti-pathogen activity of *in vivo* generated Fabs or Nbs within the *bona fide* antibody independent of the Fc part is difficult to provide as there is so far no simple assay to demonstrate such an event. Nevertheless, such mechanisms might exist as Nbs with competitive enzyme-inhibiting activity or Fabs with catalytic activity (termed Abzymes) have been identified (117–120). Under certain conditions, antibodies were documented to exert bactericidal activities in the absence of complement or phagocytes (125). For instance, antibodies catalyze the generation of hydrogen peroxide (H_2_O_2_) from singlet molecular oxygen and water, thereby producing an additional molecular species with a chemical signature similar to that of ozone (126). Interestingly, this singlet molecular oxygen is only present when the host is under assault, thereby making it an “event-triggered” substrate and consequently suggests that the additional function of an antibody might only be apparent under “inflammatory” conditions (127). In addition, it was shown that high H_2_O_2_ in concert with transition metal ions (FeCl_2_) (i.e., factors that are typically produced/released *via* apoptotic neutrophils during inflammation) can generate hydroxyl radicals (*via* a Fenton-like reaction) that induce hinge fragmentation of IgG1 mAb [(128) consisting of (i) a Fab domain and the upper hinge of one of the Fc domains and (ii) another Fab domain linked to the Fc domain (see proposed model in Figure 3)]. This indicates that under certain *in vivo* inflammatory conditions hidden biological activities could become unmasked from *bona fide* antibodies. To the best of our knowledge, the observation that an antibody-derived fragment on itself can exert a biological function is a new conceptual insight which might broaden the potentiality of Ab applications in different fields.

## Conclusion and Future Perspectives

Over the years, Nbs have been found to be valuable tools in the field of AT both at the level of diagnosis and treatment. They were shown to have potential to circumvent problems encountered with antibody-based detection systems and in addition allow the development of antigen-based approaches that were so far lacking (32). With the era of proteomics (71), allowing additional biomarkers to be discovered, the application of a Nb-based immunoproteomic approach might allow developing more efficient tools to improve trypanosomiasis control (diagnosis/treatment) in the near future. With respect to treatment against AT, it seems that Nbs are a very proficient tool to deliver drugs/toxins to parasites, thereby reducing the side effects due to drug toxicity and possibly the probability to develop drug resistance. Their small size, low immunogenicity, and tailorability furthermore favor their application in AT with respect to BBB drug delivery, routine/systemic administration, and generation of half-life extended formats. Also for research purposes, their advantages are increasingly appreciated and they will most likely become a useful tool with respect to crystallization and imaging. Finally, the concept that a Nb or a Fab fragment derived from an intact IgG might exert a biological function by itself in the absence of the Fc-bystander warrants further investigation. Moreover, this suggests that intact antibodies harbor hidden functions that only become apparent upon fragmentation into a Fab, a process that might occur under certain conditions, and this might have a broad range of implications and applications in other diseases.

## Author Contributions

BS, PB, GC, JA, JG, and SM collectively wrote the manuscript.

## Conflict of Interest Statement

The authors declare that the research was conducted in the absence of any commercial or financial relationships that could be construed as a potential conflict of interest.
